# Rift Valley fever virus virulence factor NSs targets ATF4- and XBP1-driven unfolded protein response

**DOI:** 10.1128/jvi.01233-26

**Published:** 2026-09-24

**Authors:** Kaori Terasaki, Shinji Makino

**Affiliations:** 1 Department of Microbiology and Immunology, The University of Texas Medical Branch 547647, Galveston, Texas, USA; 2 Institute for Human Infection and Immunity, The University of Texas Medical Branch 551582, Galveston, Texas, USA; 3 Center for Biodefense and Emerging Infectious Diseases, The University of Texas Medical Branch 12338, Galveston, Texas, USA; 4 UTMB Center for Tropical Diseases, The University of Texas Medical Branch 12338 , Galveston, Texas, USA; 5 The Sealy Institute for Vaccine Sciences, The University of Texas Medical Branch 559814, Galveston, Texas, USA; University of Kentucky College of Medicine, Lexington, Kentucky, USA

**Keywords:** NSs protein, unfolded protein response, Rift Valley fever virus

## Abstract

Rift Valley fever virus (RVFV) is a mosquito-borne arbovirus that infects humans and domestic ruminants, significantly impacting public health and the economy in African and Middle Eastern countries. RVFV non-structural protein NSs is a major viral virulence factor and possesses multiple functions to counteract host antiviral responses. One of its functions is to block host transcription by promoting degradation of subunits of the host transcription factor IIH (TFIIH). However, the biological significance of this function in the RVFV life cycle remains unclear. We used RVFV strain MP-12 and its mutant lacking the TFIIH degradation function (NSs-mut virus) to identify host genes targeted by NSs-mediated transcription suppression. We found that NSs-mut virus, but not MP-12, upregulated unfolded protein response (UPR) target genes compared to uninfected cells. Both viruses activated all three ER stress sensors, including PERK and IRE1. However, only MP-12 inhibited the PERK and IRE1 pathways by inhibiting the expression of their respective downstream mediators ATF4 and XBP1. ATF4 expression was inhibited at the transcriptional level, whereas XBP1 expression was inhibited at the post-transcriptional level. ATF4 depletion negatively impacted virus titer and the production of non-plaque-forming particles, resulting in alteration of the particle-to-PFU ratio of the viruses. We also showed that activation of the PERK pathway plays a pro-survival role in NSs-mut virus-infected cells, suggesting the involvement of the PERK pathway in RVFV-induced cytotoxicity. Taken together, our study revealed that RVFV NSs inhibits two out of the three UPR pathways and promotes RVFV-induced cytotoxicity by inhibiting the PERK pathway.

**IMPORTANCE:**

The Rift Valley fever virus (RVFV) NSs protein is a major virulence factor with multiple functions, including inhibition of general host transcription. RVFV carrying a mutated NSs that lacks the transcription suppression function is less cytotoxic *in vitro* and less virulent in mice compared to its parental virus, suggesting that NSs-mediated host transcription suppression is important for RVFV virulence. Experiments using this mutant virus and its parental RVFV revealed that NSs inhibits the expression of unfolded protein response (UPR) mediators ATF4 and XBP1, which act downstream of the PERK and IRE1 pathways, respectively. Our data also showed that activation of PERK promotes cell survival in RVFV infection. These data suggest that the NSs-mediated inhibition of UPR pathways contributes to RVFV pathogenicity.

## INTRODUCTION

Rift Valley fever virus (RVFV) is a zoonotic arbovirus transmitted by mosquitoes and causes outbreaks in African and Middle Eastern countries (1). The virus infects humans and domestic ruminants and causes a significant negative impact on public health and the economy. Human infection generally results in self-limiting illness, but some patients develop more severe disease, including ocular disease, encephalitis, neurological disease, and hemorrhagic fever (2–6). RVFV causes fatal disease in young animals and abortion in pregnant animals (7, 8). RVFV has the potential to spread to any area of the world via naturally occurring mosquito populations (9, 10). Furthermore, ongoing climate change may alter mosquito distribution, introducing RVFV into new areas and increasing the demand for preventatives (11, 12). While several diverse RVFV vaccine candidates have been developed (13–16), none have been licensed for human use.

RVFV belongs to the family *Phenuiviridae*, genus *Phlebovirus*, and has a three-segmented single-stranded RNA genome consisting of L, M, and S RNAs. The RVFV virion is composed of major envelope proteins Gn and Gc, viral RNA-dependent RNA polymerase (L protein), N protein, and the RNA genomes. The L RNA encodes L protein, and the M RNA encodes Gn and Gc, both of which accumulate at the ER-Golgi region, as well as two accessory proteins. The S RNA encodes the N and a non-structural protein, NSs, in an ambisense manner. Viral RNA synthesis occurs in the cytoplasm, while virus assembly takes place in the Golgi complex.

NSs is composed of 265 amino acids and has three domains: N-terminal domain, helical core domain, and C-terminal disordered domain (17). NSs localizes in both the cytoplasm and nucleus and forms filaments in the nucleus of infected cells (18). NSs is a major virulence factor and has three major functions that block host antiviral responses. First, NSs inhibits transcription of IFN-β mRNA by interacting with the repressor protein SAP30 on the promoter region of the IFN-β gene (19). Second, it promotes degradation of protein kinase R (PKR) (20–23). Third, it blocks host transcription by promoting degradation of p62, a subunit of transcription factor IIH (24–27). It is unclear whether the NSs-mediated transcription suppression broadly targets most host genes or only specific gene sets. NSs of MP-12, an attenuated RVFV strain (28), retains the same functions as the NSs protein of the wild-type (WT) RVFV (19, 22–24, 29).

We previously generated and characterized an MP-12-based recombinant virus carrying R16H and M250K mutations in the NSs (NSs-mut virus) (29). The NSs-mut virus does not cause p62 degradation and therefore does not inhibit host transcription, yet it induces PKR degradation (29). The R16H and M250K mutations do not overlap with the known functional domains of NSs that are responsible for filament formation and PKR degradation (30, 31). Analysis of the NSs-mut virus suggested that the NSs-mediated host transcription suppression partially contributes to the inhibition of IFN-β transcription (29). The study also showed that the NSs-mut virus is less cytotoxic than MP-12 in Vero E6 cells and less virulent than MP-12 in young mice (29), suggesting the contribution of the transcription suppression function of NSs to RVFV pathogenesis.

In the present study, we investigated the impact of NSs-mediated transcription suppression on the expression of host mRNAs. Transcriptome analysis of cells infected individually with MP-12 or the NSs-mut virus, followed by subsequent experiments, showed that while both viruses induced ER stress and activated all three canonical unfolded protein response (UPR) sensors, only MP-12 suppressed the expression of the transcription factors XBP1 and ATF4, which work downstream of IRE1 and PKR-like ER kinase (PERK) pathways. We also demonstrated that the activation of the PERK pathway contributed to the survival of the NSs-mut virus-infected cells.

## RESULTS

### MP-12 downregulates expression of UPR target genes

**Fig 1:**
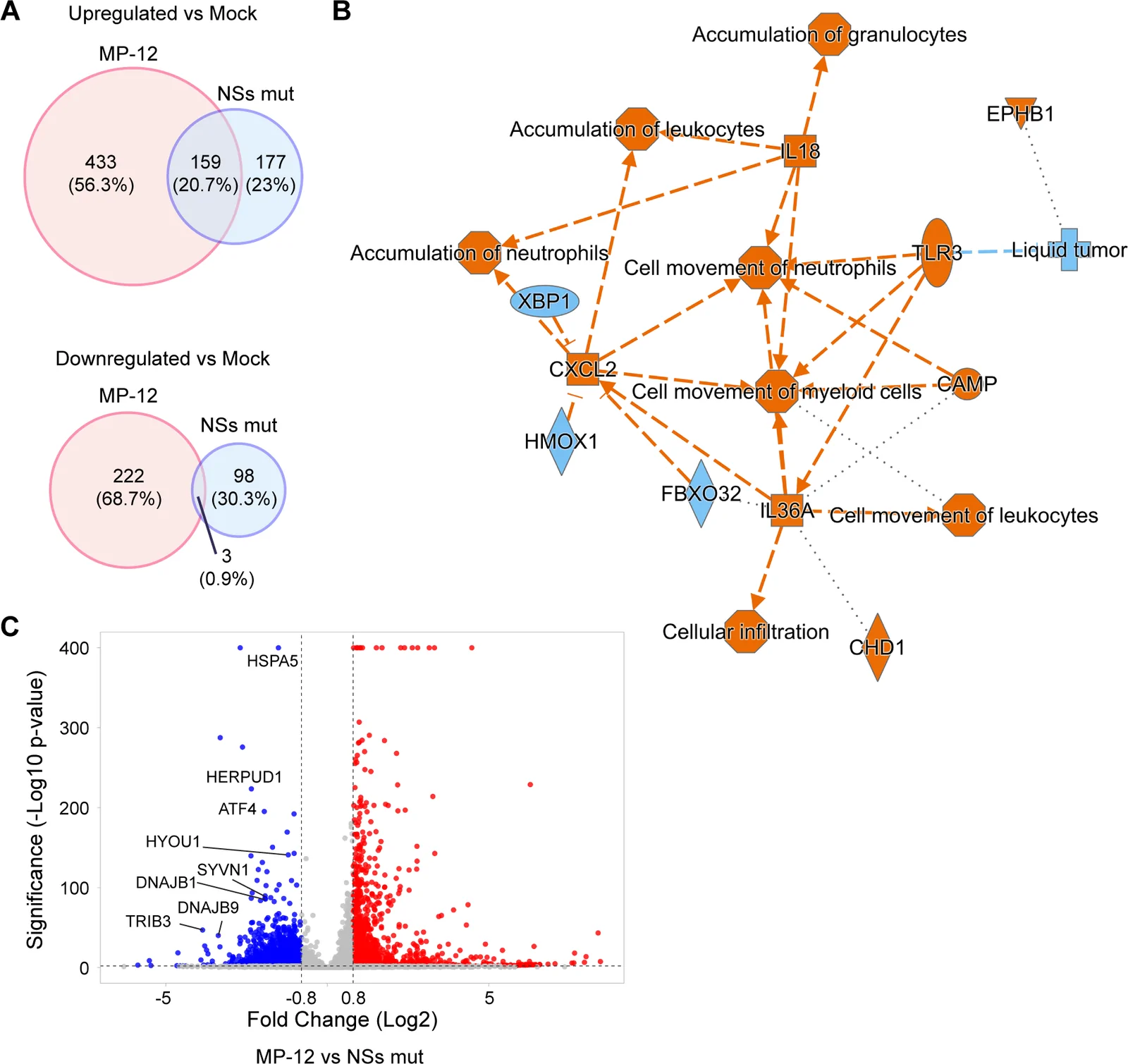
RNA-seq analysis of RVFV-infected cells. (**A**) Upregulated genes (top) and downregulated genes (bottom) in MP-12-infected cells compared to uninfected cells (red circles) and NSs-mut virus-infected cells compared to uninfected cells (blue circles) at 10 h p.i. (**B**) Network analysis between MP-12-infected cells and NSs-mut virus-infected cells. Differentially expressed genes (log_2_ fold change >1.5) in MP-12-infected cells compared to NSs-mut virus-infected cells were analyzed by using Qiagen IPA. In MP-12-infected cells, orange indicates upregulation and blue indicates downregulation of pathways compared to NSs-mut virus-infected cells. (**C**) Volcano plot of gene expression changes (log_2_ fold change >0.8) in MP-12-infected cells compared to NSs-mut virus-infected cells. The names of UPR-related genes are indicated.

To investigate the effects of NSs-mediated transcription suppression on the expression of various host mRNAs, we performed transcriptome analysis of cells infected with MP-12 or the NSs-mut virus. The NSs-mut virus induces higher levels of IFN-β mRNA than MP-12 in MRC-5 cells (29). To exclude the effect of IFN-β on host gene expression, we used Vero E6 cells, which lack the type I IFN genes, for virus preparation and transcriptome analysis. For the transcriptome analysis, we prepared total intracellular RNAs from infected cells at a late stage of infection (10 h post-infection [p.i.]). Viral RNA accumulation levels at 6 and 10 h p.i. were similar between MP-12 and NSs-mut virus-infected cells (Fig. S1). Poly(A)-containing RNAs were subjected to next-generation sequencing. As viral mRNAs are not polyadenylated (32), this experimental approach enriches host mRNAs. The total read counts of the MP-12- and NSs-mut virus-infected samples were 11% and 5% lower, respectively, than the mock-infected samples. Among the differentially expressed genes, 592 genes and 225 genes were upregulated and downregulated, respectively, in MP-12-infected cells (Fig. 1A), while NSs-mut virus-infected cells showed 336 upregulated genes and 101 downregulated genes. Of the differentially expressed genes, 20.7% of the upregulated genes and 0.9% of the downregulated genes were common between MP-12-infected cells and NSs-mut virus-infected cells.

The top 10 downregulated genes in MP-12-infected cells compared to NSs-mut virus-infected cells included HSPA5, HERPUD1, and ATF4 (Table 1). These genes are associated with the UPR, a cellular stress response induced by ER stress. HSPA5 and HERPUD1 genes were significantly upregulated in NSs-mut virus-infected cells compared to mock-infected cells (Table S1). These two genes were also upregulated in MP-12-infected cells compared to mock-infected cells, although the difference was below the cutoff value (log_2_ fold change <1.5). These data showed that NSs-mut virus infection increased the expression of HSPA5 and HERPUD1 genes to a greater extent than MP-12 infection. The top 10 upregulated genes in MP-12-infected cells compared to NSs-mut virus-infected cells included NFKBIZ, TNFAIP3, and NFKBIA (Table 1), all of which are associated with the NF-κB signaling pathway (33). The NSs-mut virus also induced higher levels of the TNFAIP3 and NFKBIA genes, albeit to a lesser extent than MP-12 (Table S1). Although we used Vero E6 cells for transcriptome analysis, both viruses induced several interferon-stimulated genes (ISGs) (34) (Table S2).

**TABLE 1:** Top 10 up- and downregulated genes in MP-12-infected cells compared to NSs-mut virus-infected cells

	Gene symbol	Log_2_ fold change	Gene name
Up	NFKBIZ	4.5	NFKB inhibitor zeta
	TNFAIP3	3.3	TNF alpha-induced protein 3
	NUAK2	3.2	NUAK family kinase 2
	AMOTL2	2.8	Angiomotin-like 2
	CCN1	2.6	Cellular communication network factor 1
	EPHA2	2.4	EPH receptor A2
	MAP3K1	2.3	Mitogen-activated protein kinase kinase kinase 1
	PLK2	1.7	Polo-like kinase 2
	LAMA4	1.5	Laminin subunit alpha 4
	NFKBIA	1.8	NFKB inhibitor alpha
Down	HSPA1A	−2.7	Heat shock protein family A (Hsp70) member 1A
	HSPA5	−1.5	Heat shock protein family A (Hsp70) member 5
	CLDN4	−3.3	Claudin 4
	HMOX1	−2.6	Heme oxygenase 1
	HERPUD1	−2.3	Homocysteine-inducible ER protein with ubiquitin-like domain 1
	ATF4	−1.9	Activating transcription factor 4
	SEC24D	−1.7	SEC24 homolog D, COPII coat complex component
	BAHCC1	−2.4	BAH domain and coiled-coil containing 1
	BIRC2	−2	Baculoviral IAP repeat containing 2
	NUFIP2	−2.1	Nuclear FMR1-interacting protein 2

Network analysis of differentially expressed genes between MP-12-infected cells and NSs-mut virus-infected cells suggested inhibition of three genes, including XBP1, HMOX1, and FBXO32, in MP-12-infected cells (Fig. 1B). The former two genes are closely related to UPR (35, 36). The expression levels of HMOX1 (Table 1), XBP1, and FBXO32 genes were lower in MP-12-infected cells than in NSs-mut virus-infected cells, although the differences were smaller than the cutoff value for the latter two genes (−1.216 for XBP1 and −1.028 for FBXO32). Liquid tumor was identified as a significantly inhibited disease in MP-12-infected cells, compared to NSs-mut virus-infected cells. Volcano plot analysis showed downregulation of several ER stress-related genes (37, 38) in MP-12-infected cells as compared to NSs-mut virus-infected cells (Fig. 1C). Ingenuity pathway analysis (IPA) also showed that UPR was one of the significantly inhibited pathways in MP-12-infected cells compared to NSs-mut virus-infected cells (Table 2). Table 3 lists the differentially expressed UPR-related genes between MP-12-infected cells and NSs-mut virus-infected cells that were identified by the IPA. Taken together, these results suggest that MP-12 inhibited UPR-mediated gene expression.

**TABLE 2:** Up- and downregulated pathways in MP-12-infected cells vs NSs-mut virus-infected cells

Ingenuity canonical pathway	−Log(*P* value)	*z* score
Cardiac hypertrophy signaling	2.28	2.828
Production of nitric oxide and reactive oxygen species in macrophages	2.18	2
Dilated cardiomyopathy signaling pathway	2.99	−2.496
Unfolded protein response	3.11	−2.53

**TABLE 3:** The log_2_ fold change of the top 10 differentially expressed UPR target genes identified by pathway analysis and other potential ATF4 target genes

Gene	Log_2_ FC vs mock		Protein	Function	Upstream regulator in NSs-mut
	MP-12	NSs-mut			
HSPA5	1.0	2.5	Heat shock protein (HSP) 70-A5 (BiP)	Chaperone	XBP1
ATF4	−1.1	0.8	Activating transcription factor 4	Transcription factor	–^*a*^
SYVN1	0	1.9	Synoviolin 1	E3 ubiquitin ligase	XBP1
DNAJB1	−0.4	1.5	HSP40-B1	Chaperone	–
DNAJC3	0.4	1.9	HSP40-C3	Chaperone	–
CEBPB	−1.6	0.5	CCAAT enhancer binding protein (C/EBP)-beta	Transcription factor	XBP1
DNAJB9	0	3.4	HSP40-B9	Chaperone	–
TRAF2	−0.8	1.1	TNF receptor associated factor 2	E3 ubiquitin ligase	–
CEBPG	−1.2	1.1	C/EBP-gamma	Transcription factor	–
ERO1B	−0.1	1.6	ER oxidoreductase 1 beta	Oxidoreductase	–
HMOX1	−0.7	1.9	Heme oxygenase 1	Enzyme	ATF4
PPP1R15A	2.4	2.3	Growth arrest and DNA damage-inducible protein 34 (GADD34)	Dephosphorylation of eIF2α	Unknown
DDIT3	2.1	2.8	C/EBP homologous protein (CHOP)	Transcription factor	Unknown

^*a*^ –, not tested.

### MP-12 and NSs-mut virus activate three ER stress sensors, while only MP-12 inhibits expression of UPR mediators

**Fig 2:**
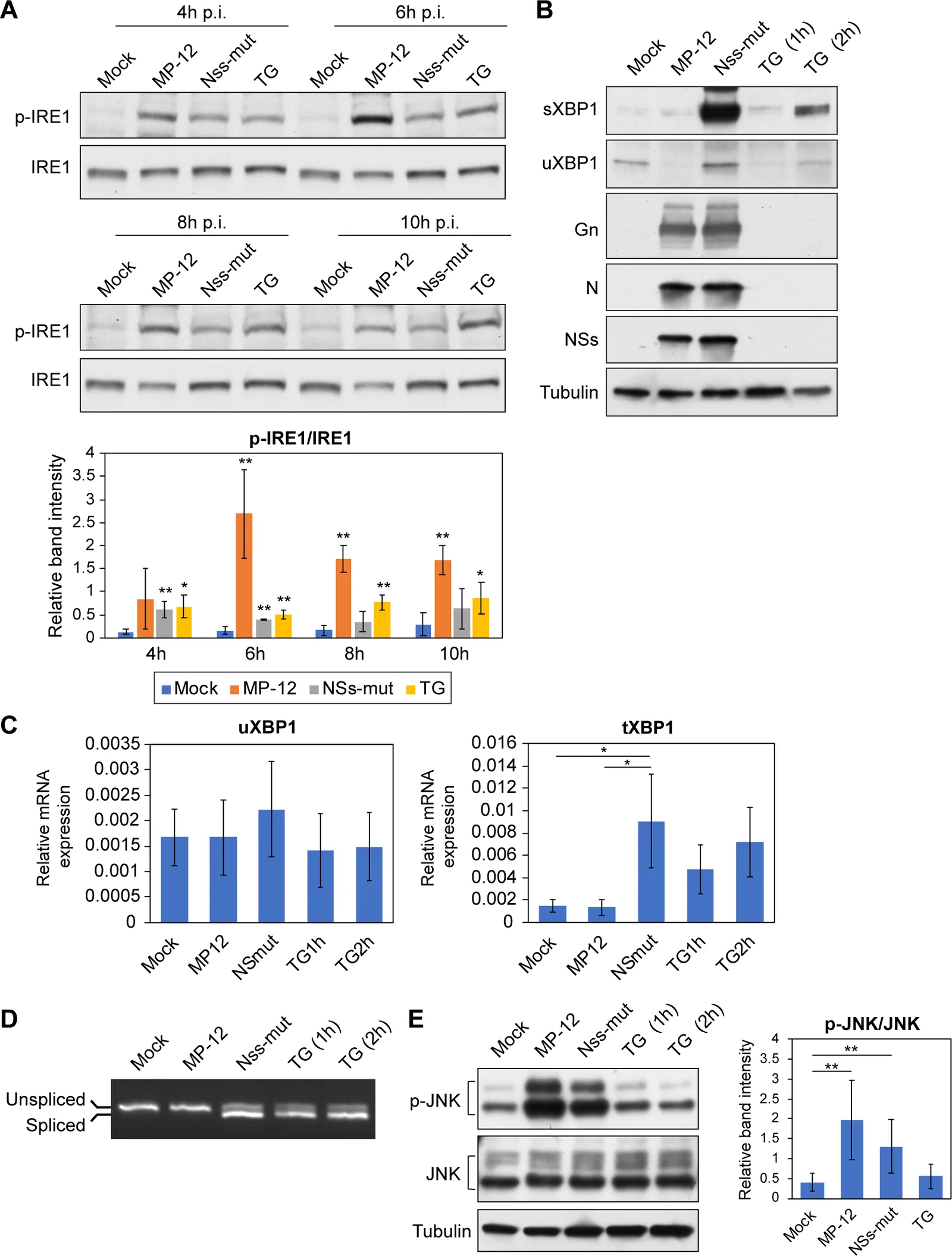
Inhibition of XBP1 expression in MP-12-infected cells. (**A**) Phosphorylation of IRE1. Vero E6 cells were infected with the indicated virus at an MOI of 3. Uninfected cells and cells treated with 400 nM of TG for 1 h were used as controls. Cell extracts were harvested at indicated points and analyzed by Western blot using antiphospho-IRE1 and anti-IRE1 antibodies. The p-IRE1/IRE1 ratio was calculated by dividing the band intensity of p-IRE1 by the band intensity of IRE1. (**B**) Expression of sXBP1 and uXBP1. Vero E6 cells were infected with the indicated virus at an MOI of 3. Uninfected cells and cells treated with TG for 1 and 2 h were used as controls. Cell lysates were harvested at 6 h p.i. and analyzed by Western blot using anti-XBP1 antibody, which detects both sXBP1 and uXBP1, antibodies against viral proteins, and antitubulin antibody. (**C**) RT-qPCR analysis of unspliced XBP1 mRNA (uXBP1) and total XBP1 mRNA (tXBP1). Total RNA was extracted from virus-infected cells and control cells at 6 h p.i. and subjected to RT-qPCR. (**D**) Splicing of XBP1 mRNA. Total RNA was extracted from infected cells and control cells at 6 h p.i. and subjected to PCR using a primer set that detects both unspliced and spliced XBP1 mRNAs. (**E**) Phosphorylation of JNK. Vero E6 cells were infected with the indicated virus at an MOI of 3 or treated with TG. Cell lysates were harvested at 6 h p.i. and analyzed by Western blot using antibodies against phosphor-JNK, JNK, and tubulin. The p-JNK/JNK ratio was calculated by dividing the band intensity of p-JNK by the band intensity of JNK. *P < 0.05, **P < 0.01.

UPR is activated by three ER stress sensors, including inositol-requiring enzyme 1 (IRE1), PERK, and activating transcription factor 6 (ATF6) (39). ER stress induces IRE1 autophosphorylation, which promotes expression of the XBP1 protein (40, 41). To investigate activation of the IRE1-XBP1 axis in infected cells, we first determined the ratio of phosphorylated-IRE1 (p-IRE1) to total IRE1 at various times p.i. Thapsigargin (TG), a chemical ER stress inducer, was used as a positive control. Both viruses induced a higher p-IRE1/total IRE1 ratio compared to mock-infected cells as early as 6 h p.i. (Fig. 2A), demonstrating that RVFV infection activates IRE1.

Phosphorylated IRE1 induces cleavage of XBP1 mRNA through its RNase activity (42), resulting in the generation of a spliced form of XBP1 mRNA. The spliced form of XBP1 mRNA encodes sXBP1, which is a transcription factor regulating UPR, while the unspliced XBP1 mRNA encodes uXBP1, which interacts with the p53 pathway to modulate cell cycle processes (43). The NSs-mut virus induced higher expression levels of sXBP1 and uXBP1 than MP-12 at 6 h p.i. (Fig. 2B), indicating that NSs inhibits XBP1 expression. We noted the inhibition of XBP1 expression as early as 4 h p.i. in MP-12-infected cells (Fig. S2A). The levels of unspliced XBP1 mRNA were not significantly different among mock-infected cells, MP-12-infected cells, NSs-mut virus-infected cells, and TG-treated cells (Fig. 2C, uXBP1), indicating that MP-12 did not inhibit transcription of XBP1 mRNA. The total XBP1 mRNA levels, including both unspliced and spliced forms, increased in NSs-mut virus-infected cells but not in MP-12-infected cells (Fig. 2C, tXBP1), suggesting the absence of the spliced form of XBP1 mRNA in MP-12-infected cells. Indeed, RT-PCR analysis revealed the presence of the spliced form of XBP1 mRNA in NSs-mut virus-infected cells but not in MP-12-infected cells and uninfected cells (Fig. 2D). These results suggest that MP-12 inhibited sXBP1 expression by suppressing splicing of XBP1 mRNA.

Because NSs inhibits nuclear export of host mRNA (44) and XBP1 mRNA splicing occurs in the cytoplasm (45), NSs-induced inhibition of mRNA export could be the mechanism for the inhibition of XBP1 mRNA splicing in MP-12-infected cells. To test this possibility, we examined the status of mRNA export inhibition at the early stages of infection by counting the number of cells showing nuclear staining of polyadenylate binding protein 1 (PABP1) (Fig. S2B), as previously reported (44). PABP1 is usually found in the cytoplasm, and its abnormal presence in the nucleus reflects the accumulation of polyadenylated mRNAs trapped there. At 4 h p.i., the number of cells showing nuclear PABP1 staining was similar between mock-infected cells and MP-12-infected cells (Fig. S2C). The number of cells showing nuclear PABP1 staining started increasing at 6 h p.i. in MP-12-infected cells, suggesting that the inhibition of mRNA export starts around 6 h p.i. In contrast, prominent nuclear accumulation of PABP1 did not occur in NSs-mut virus-infected cells, suggesting absence of mRNA export inhibition. The results suggest that XBP1 inhibition in MP-12-infected cells occurred prior to the NSs-mediated mRNA export inhibition. These data suggest that the NSs-induced inhibition of mRNA export may not be the mechanism of the NSs-mediated inhibition of XBP1 mRNA splicing.

In addition to an RNase domain, IRE1 carries a kinase domain (46). Activation of the kinase domain causes phosphorylation of c-Jun N-terminal kinase (JNK) through interaction with E3 ubiquitin ligase TRAF2 (47). Since our data indicated that MP-12 inhibits the RNase activity of IRE1, we next examined whether MP-12 also inhibits the kinase function of IRE1, leading to reduced JNK phosphorylation compared with the NSs-mut virus. Western blot analysis of the cell extracts from mock-, MP-12-, and NSs-mut virus-infected cells and TG-treated cells showed multiple JNK bands, which represent products from spliced forms of JNK mRNA (48). Since the short JNK variant was dominant in this cell line, we measured the band intensity of the short variant. Replication of both viruses resulted in a higher ratio of phosphorylated JNK to total JNK than in uninfected cells, demonstrating induction of JNK phosphorylation by RVFV replication (Fig. 2E). The two viruses induced similar levels of JNK phosphorylation, suggesting that MP-12 did not inhibit the kinase activity of IRE1 and/or other pathways that induce JNK phosphorylation. Short-term treatment with TG did not induce JNK phosphorylation in this cell line (Fig. 2E).

**Fig 3:**
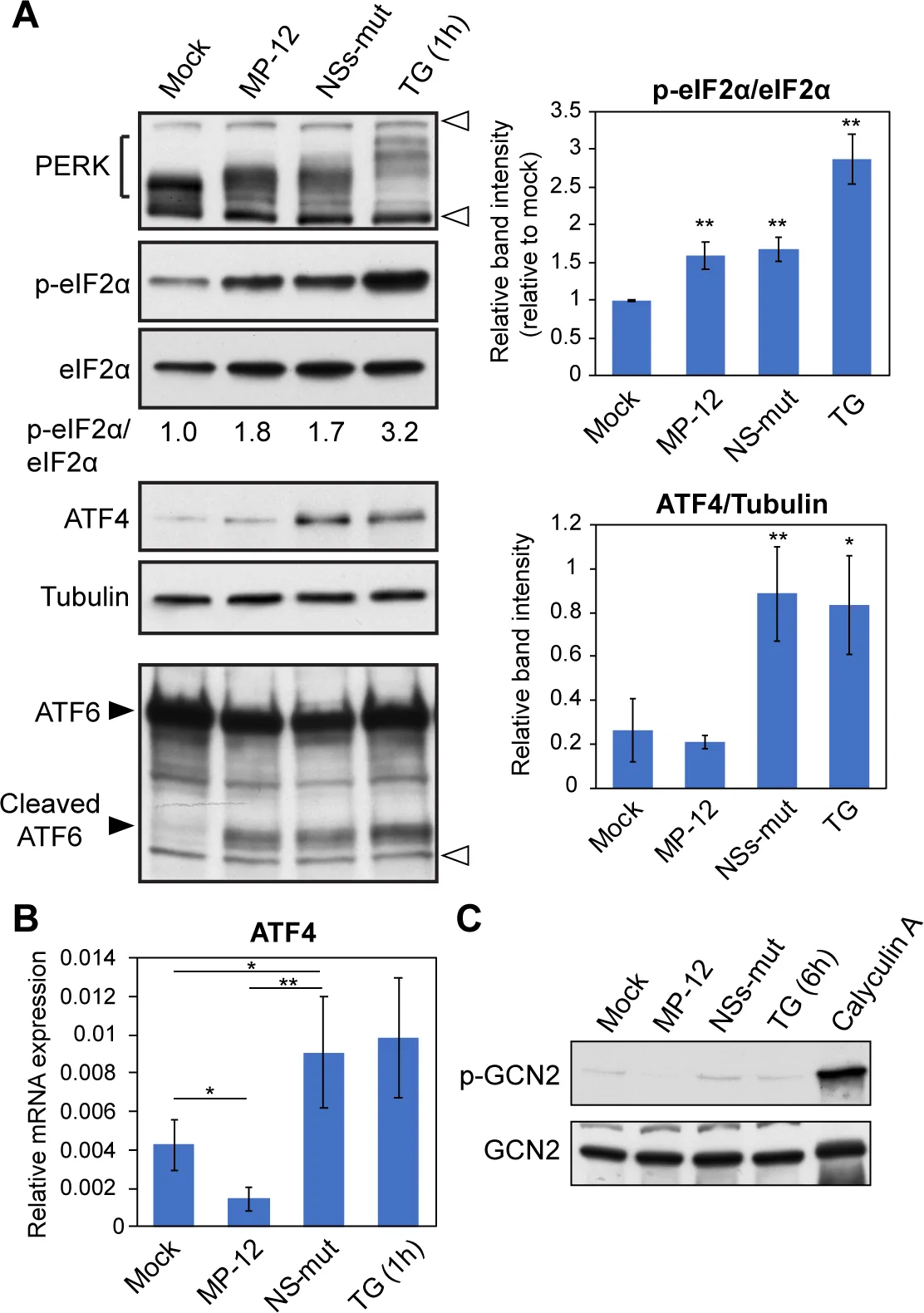
Analysis of PERK and ATF6 arms of the UPR in RVFV-infected cells. (**A**) Activation of PERK and ATF6 pathways. Vero E6 cells were mock-infected or infected with the indicated virus at an MOI of 3 or treated with TG. Cell extracts were harvested at 6 h p.i. and analyzed by Western blot by using antibodies against the indicated proteins. Open arrowheads represent non-specific signals. The p-eIF2-α/eIF2α and ATF4/tubulin ratios were calculated by using band intensities of these proteins in the Western blot analyses. (**B**) RT-qPCR analysis of ATF4 mRNA. Total RNA was extracted from virus-infected cells and control cells at 6 h p.i. and subjected to RT-qPCR. (**C**) Activation of GCN2. Infection was performed as described in panel A. Cells treated with 100 nM of calyculin A were used as a control. Cell extracts were harvested at 6 h p.i. and analyzed by Western blot using GCN2 and phospho-GCN2 antibodies. *P < 0.05, **P < 0.01.

PERK has multiple phosphorylation sites and undergoes trans-autophosphorylation upon induction of ER stress (49). Phosphorylated PERK induces the phosphorylation of eIF2α, leading to attenuation of global protein synthesis while promoting ATF4 translation via bypass of upstream open reading frames in the ATF4 mRNA (50). Western blot analysis revealed higher levels of slower-migrating PERK signals in both MP-12- and NSs-mut virus-infected cells than in mock-infected cells (Fig. 3A). Since these slower-migrating PERK signals represent phosphorylated PERK (51), the data indicated that both viruses induced PERK phosphorylation. Both viruses induced higher levels of phosphorylated eIF2α compared to mock-infected cells, whereas only NSs-mut virus induced ATF4 expression (Fig. 3A). RT-qPCR analysis further showed that ATF4 mRNA level was significantly lower in MP-12-infected cells than in NSs-mut virus-infected cells and uninfected cells (Fig. 3B). These data showed that both MP-12 and NSs-mut virus infection induced PERK phosphorylation and eIF2α phosphorylation, whereas only MP-12 inhibited ATF4 expression at the transcriptional level.

There are other kinases besides PERK that can induce eIF2α phosphorylation; these kinases include PKR, general control nonderepressible 2 (GCN2), and heme-regulated inhibitor (HRI) (52). However, neither PKR nor HRI is likely a significant contributor to the eIF2α phosphorylation in RVFV-infected cells, as both MP-12 and NSs-mut virus induce PKR degradation (29), and HRI is primarily expressed in erythroid cells (52). Accordingly, we next examined whether GCN2 is also involved in eIF2α phosphorylation in RVFV-infected cells. Since phosphorylation of GCN2 is required for its kinase activity (53), we examined the phosphorylation status of GCN2 during infection. Cells treated with calyculin A, a phosphatase inhibitor, showed efficient phosphorylation of GCN2, whereas TG treatment did not induce GCN2 phosphorylation (Fig. 3C). Phosphorylation levels of GCN2 in MP-12-infected cells and NSs-mut virus-infected cells were similar to those in mock-infected cells (Fig. 3C), demonstrating that RVFV infection did not induce GCN2 phosphorylation in Vero E6 cells.

Lastly, we examined the activation status of the third ER stress sensor, ATF6. ER stress induces translocation of ATF6 from the ER to the Golgi, where ATF6 is cleaved by site one protease (54, 55). Both viruses and TG treatment induced efficient cleavage of ATF6, while mock-infected cells showed a very low level of cleaved ATF6 (Fig. 3A, bottom panel), suggesting the ATF6 pathway is activated by both viruses.

Taken together, both MP-12 and NSs-mut virus activated three ER stress sensors, yet only MP-12 inhibited the expression of two major UPR mediators, XBP1 and ATF4, which act downstream of the IRE1 and PERK pathways, respectively.

### ATF4 depletion reduces RVFV particle production

**Fig 4:**
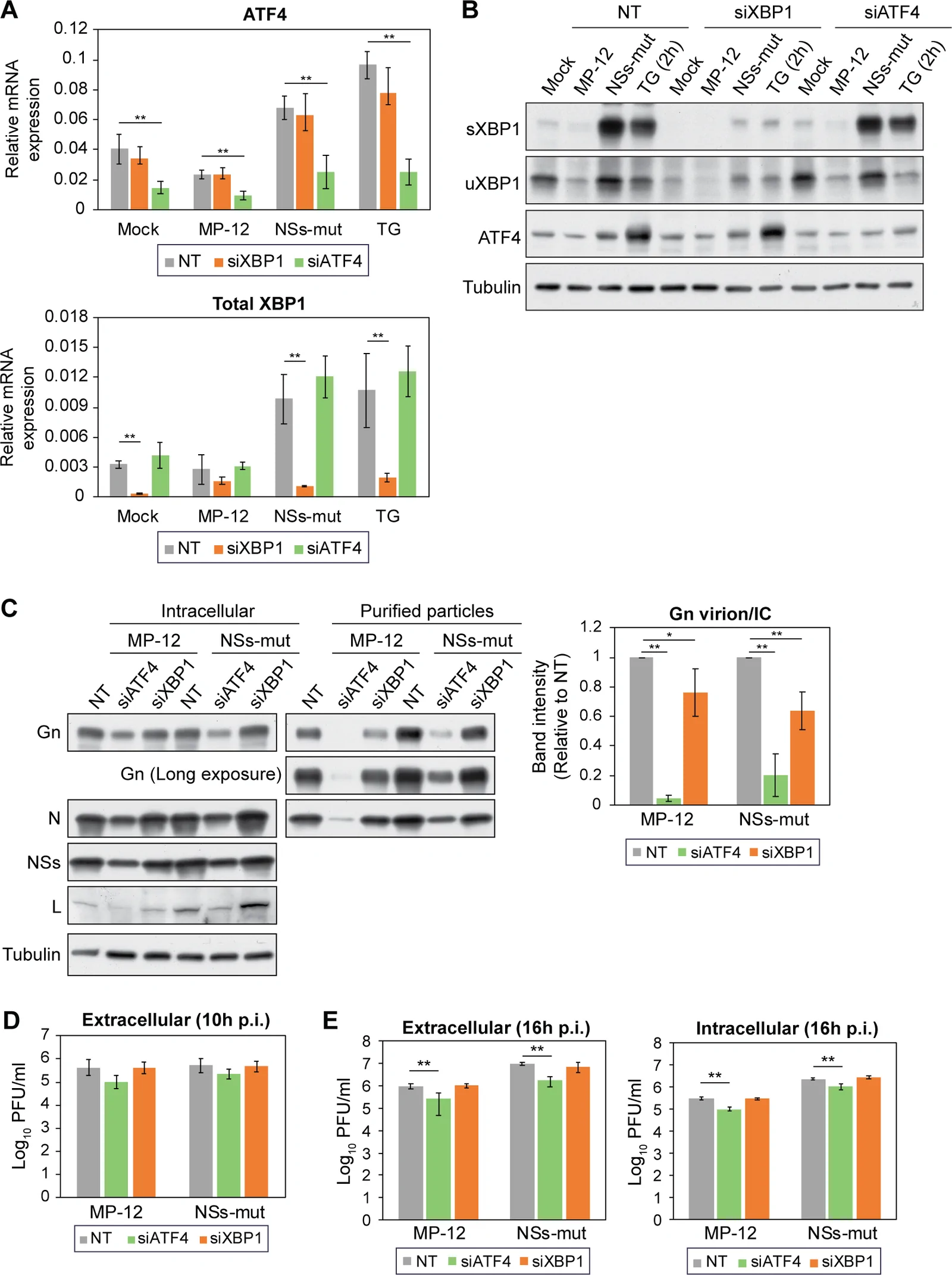
The effects of ATF4 or XBP1 depletion on virus production. (**A**) ATF4 and XBP1 mRNA levels in XBP1- or ATF4-depleted cells. Vero E6 cells were transfected with non-targeting (NT) siRNA, siXBP1 RNA, or siATF4 RNA and infected with the indicated virus at an MOI of 0.5 at 48 h post transfection. Cells transfected with siRNA and treated with TG for 2 h were used as a control. Total RNA was extracted from the cells at 10 h p.i. and subjected to RT-qPCR. (**B**) Intracellular accumulation of sXBP1, uXBP1, and ATF4 in XBP1- or ATF4-depleted cells. Transfection and infection were performed as described in panel A. Cell extracts were harvested at 10 h p.i. and analyzed by Western blot using antibodies against the proteins listed on the left side of the panels. (**C**) Viral protein accumulation in siRNA-transfected cells. siRNA transfection and infection were performed as described in panel A. Cells were harvested at 10 h p.i. and analyzed by Western blot using antibodies against viral proteins and tubulin. RVFV particles were purified from culture supernatant harvested at 10 h p.i. by sucrose gradient ultracentrifugation and analyzed by Western blot. Gn virion/IC ratio was calculated by dividing Gn band intensity in purified samples by Gn band intensity in intracellular samples. (**D**) Extracellular infectious virus titers in siRNA-transfected cells at 10 h p.i. siRNA transfection and infection were performed as described in panel A. Culture supernatant was harvested at 10 h p.i. and subjected to a plaque assay. (**E**) Extracellular and intracellular virus titers in siRNA-transfected cells at 16 h p.i. siRNA transfection and infection were performed as described in panel A. Culture supernatant was harvested at 16 h p.i. and subjected to a plaque assay. To determine intracellular infectious viruses, infected cells were harvested after washing and subjected to three freeze and thaw cycles. Supernatant was subjected to a plaque assay after removing cell debris by centrifugation. *P < 0.05, **P < 0.01.

To explore the biological significance of MP-12-mediated inhibition of XBP1 and ATF4 expression, the effects of siRNA-mediated depletion of ATF4 and XBP1 on virus replication were investigated. Since ATF4 is a stress-inducible gene and its protein expression is controlled at the post-transcriptional level, we performed RT-qPCR analysis to evaluate the effect of siATF4 on ATF4 mRNA levels under stress conditions (TG treatment) (Fig. 4A). Expression of ATF4 mRNA was significantly decreased in siATF4-transfected cells compared to non-targeting siRNA (NT siRNA)-transfected cells in the presence of TG, confirming ATF4 was depleted by the siATF4 transfection. ATF4 mRNA levels in non-stressed (mock) and RVFV-infected cells also decreased following the siATF4 transfection. Expression of total XBP1 mRNAs, which includes both sXBP1 and uXBP1 mRNAs, was significantly decreased by transfection of siXBP1 targeting the uXBP1 gene (Fig. 4A). As expected, transfection with siXBP1 or siATF4 reduced the expression levels of uXBP1 and ATF4 proteins, respectively, in NSs-mut virus-infected cells and TG-treated cells (Fig. 4B). The expression level of sXBP1 was also reduced by siXBP1 transfection in NSs-mut virus-infected cells and TG-treated cells because sXBP1 mRNA is derived from uXBP1 mRNA. sXBP1 and ATF4 protein levels in MP-12-infected cells were similar to mock-infected cells in all conditions due to NSs-induced inhibition of XBP1 splicing (Fig. 2D) and ATF4 transcription inhibition (Fig. 3B).

To examine the effect of ATF4 and XBP1 depletion on RVFV production, cells were transfected with NT siRNA, siXBP1, or siATF4 and subsequently infected with MP-12 or NSs-mut virus. The cell extracts and supernatant were harvested at 10 h p.i. The supernatant was subjected to purification of virus particles and plaque assay. In MP-12 and NSs-mut virus-infected cells, XBP1 depletion had an insignificant effect on intracellular accumulation of viral proteins, whereas ATF4 depletion reduced the intracellular accumulation of viral proteins (Fig. 4C, left panels). To evaluate the effect of ATF4 or XBP1 depletion on the efficiency of virion production, the amount of Gn in purified virions was normalized by intracellular Gn accumulation levels. Depletion of ATF4 and XBP1 significantly reduced the Gn signals in the purified virion samples compared to those in infected cells (Fig. 4C, right panel). These data suggest that ATF4 and XBP1 depletion significantly reduced the production of virus particles. Especially, ATF4 depletion caused an 80%–95% reduction in virion production. ATF4 depletion resulted in a reduction of approximately 50%–80% in the infectious virus titers of MP-12 and NSs-mut viruses, whereas XBP1 depletion had no effect on the production of infectious virus titers (Fig. 4D). Although these results are noteworthy, they were deemed statistically insignificant due to the high level of variance between replicate samples. Nonetheless, the greater reduction in virus particle production compared to infectious virus titers in ATF4-depleted cells suggested that ATF4 depletion altered the particle-to-PFU ratio of the viruses. To further investigate whether virus particle release or assembly is inhibited by ATF4 depletion, we determined both intracellular and extracellular infectious virus titers at 16 h p.i. Depletion of ATF4 significantly reduced both the intracellular and extracellular infectious virus titers (Fig. 4E), suggesting that ATF4 depletion had a negative impact on the viral assembly process.

### XBP1 and ATF4 regulate expression of several UPR-induced genes in infected cells

**Fig 5:**
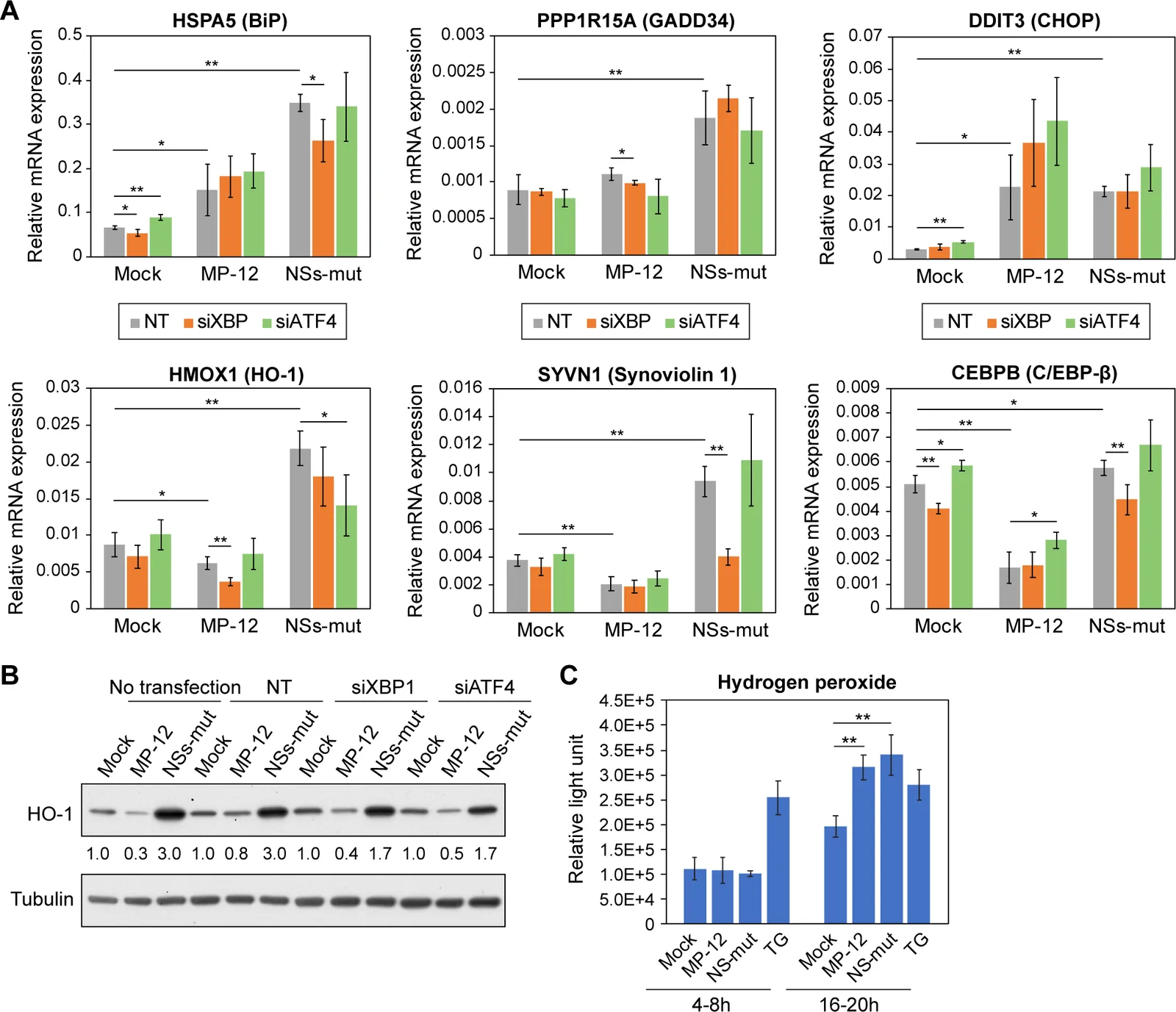
Expression of UPR-related genes in XBP1- or ATF4-depleted cells. (**A**) RT-qPCR analysis of UPR-related genes. Vero E6 cells were transfected with non-targeting (NT) siRNA, siXBP1 RNA, or siATF4 RNA and infected with the indicated virus at an MOI of 3 at 48 h post-transfection. Cells without siRNA transfection (no transfection) were used as controls. Cell lysates were harvested at 10 h p.i. Total RNA was extracted from the cells and subjected to RT-qPCR. (**B**) HO-1 expression in XBP1-depleted cells and ATF4-depleted cells. Transfection and infection were performed as described in panel A. Cell lysates were harvested at 16 h p.i. and analyzed by Western blot using anti-HO-1 and antitubulin antibodies. (**C**) Hydrogen peroxide levels in infected cells. Vero E6 cells were infected with the indicated virus at an MOI of 3 and treated with H_2_O_2_ substrate 4 h prior to the detection of luminescence. Luminescence was measured at 8 and 20 h p.i. *P < 0.05, **P < 0.01.

As XBP1 and ATF4 are transcription factors that regulate the UPR and the NSs-mut virus induces the expression of these proteins, it is likely that XBP1 or ATF4 are responsible for the upregulation of several UPR-related genes in NSs-mut virus-infected cells (Table 3). We next investigated the effect of depleting ATF4 or XBP1 on the expression of several UPR-related genes in RVFV-infected cells (Fig. 5A). Cells were transfected with NT siRNA, siXBP1, or siATF4 or left untransfected. Subsequently, these cells were infected with either MP-12 or NSs-mut virus or mock-infected. Expression levels of the following genes at 10 h p.i. were examined by RT-qPCR: HSPA5, SYVN1, CEBPB, DDIT3, PPP1R15A, and HMOX1. The fold changes of these genes in our transcriptome analysis are shown in Tables 1 and 3.

The data from RT-qPCR analysis of NT siRNA-transfected cells generally supported the RNA-seq analysis results for most of these genes, shown in Table 3, though we noted two exceptions, PPP1R15A and SYVN1. Although the RNA-seq analysis showed that MP-12 induced PPP1R15A upregulation compared to mock-infected cells, MP-12 did not upregulate PPP1R15A in NT siRNA-transfected cells (Fig. 5A). The RNA-seq analysis showed that MP-12 replication did not change SYVN1 gene levels compared to mock-infected cells, whereas RT-qPCR analysis showed that MP-12 downregulated SYVN1 gene in NT siRNA-transfected cells (Fig. 5A).

XBP1 depletion using siRNA resulted in reduced PPP1R15A and HMOX1 levels in MP-12-infected cells (Fig. 5B), suggesting that XBP1 plays a role in the expression of these genes during MP-12 infection. Depletion of XBP1 also significantly decreased the levels of HSPA5 and CEBPB in both mock-infected and NSs-mut virus-infected cells, as well as the level of SYVN1 in NSs-mut virus-infected cells. These data suggest that XBP1 plays a role in the upregulation of these genes. We noted that HSPA5 expression was higher in NSs-mut virus-infected cells than in mock-infected cells even under XBP1 depletion conditions. This indicates that other transcription factors are involved in the upregulation of HSPA5 in NSs-mut virus-infected cells.

The expression level of CEBPB was higher in siATF4-transfected MP-12-infected cells than in NT siRNA-transfected MP-12-infected cells. Similar results were also observed in mock-infected cells. However, ATF4 depletion did not affect the expression level of CEBPB in NSs-mut virus-infected cells. These data suggest that ATF4 negatively regulates CEBPB during MP-12 infection. In NSs-mut virus-infected cells, ATF4 depletion significantly reduced HMOX1 levels compared to NT siRNA-transfected cells, suggesting that ATF4 contributes to the upregulation of HMOX1 expression. DDIT3 expression levels were not affected by XBP1 or ATF4 depletion in NSs-mut virus-infected cells, suggesting that expression of DDIT3 is regulated by other transcription factors.

Taken together, the experiments using siXBP1 and siATF4 revealed the importance of XBP1 and ATF4 in the expression of UPR-related genes in RVFV infection. The roles of ATF4 and XBP1 differed between MP-12- and NSs-mut virus-infected cells, suggesting that these proteins may have redundant functions and that their roles are context dependent.

### Analysis of HO-1 expression in RVFV-infected cells

Network analysis showed that MP-12 inhibited the pathway related to the HMOX1 gene (Fig. 1B), which encodes an antioxidant HO-1. As HO-1 can regulate reactive oxygen species (ROS) production, which is induced by RVFV infection (56), we investigated the role of ATF4 and XBP1 in HO-1 expression in infected cells. Cells that were either untransfected or transfected with NT siRNA, siXBP1, or siATF4 were mock-infected or infected with either MP-12 or NSs-mut virus. Cell extracts were collected at 16 h p.i. HO-1 protein levels in MP-12-infected cells were lower than those in NSs-mut virus-infected cells in all conditions (Fig. 5B), even though there was no significant difference in HMOX1 mRNA levels between NSs-mut virus-infected cells and MP-12-infected cells in siATF4-transfected cells (Fig. 5A). These data suggest that HO-1 expression was regulated at a post-transcriptional level by other factors in addition to ATF4-mediated transcriptional regulation in infected cells.

We further investigated whether the NSs-mediated inhibition of HO-1 expression contributes to ROS production in infected cells by measuring levels of hydrogen peroxide (H_2_O_2_), one form of ROS (57). Both MP-12 and NSs-mut virus induced similarly elevated levels of H_2_O_2_ at 16–20 h p.i., suggesting that HO-1 expression level was not the major determinant of ROS level in RVFV-infected cells (Fig. 5C).

### PERK phosphorylation has a pro-survival effect in NSs-mut virus infection

Others reported that the PERK-ATF4 axis induces apoptotic cell death under persistent ER stress by upregulating the expression of DDIT3 mRNA, which encodes CHOP (58). The NSs-mut virus induced a higher level of ATF4 expression than MP-12 (Fig. 3) and expressed DDIT3 at a similar level to MP-12 (Fig. 5A), yet the NSs-mut virus is less cytotoxic than MP-12 (29). As our data did not align well with the reported pro-apoptotic effects of the PERK-ATF4-CHOP axis, we used the PERK inhibitor GSK2606414 (GSK414) to investigate the role of the PERK pathway in the death and survival of RVFV-infected cells.

**Fig 6:**
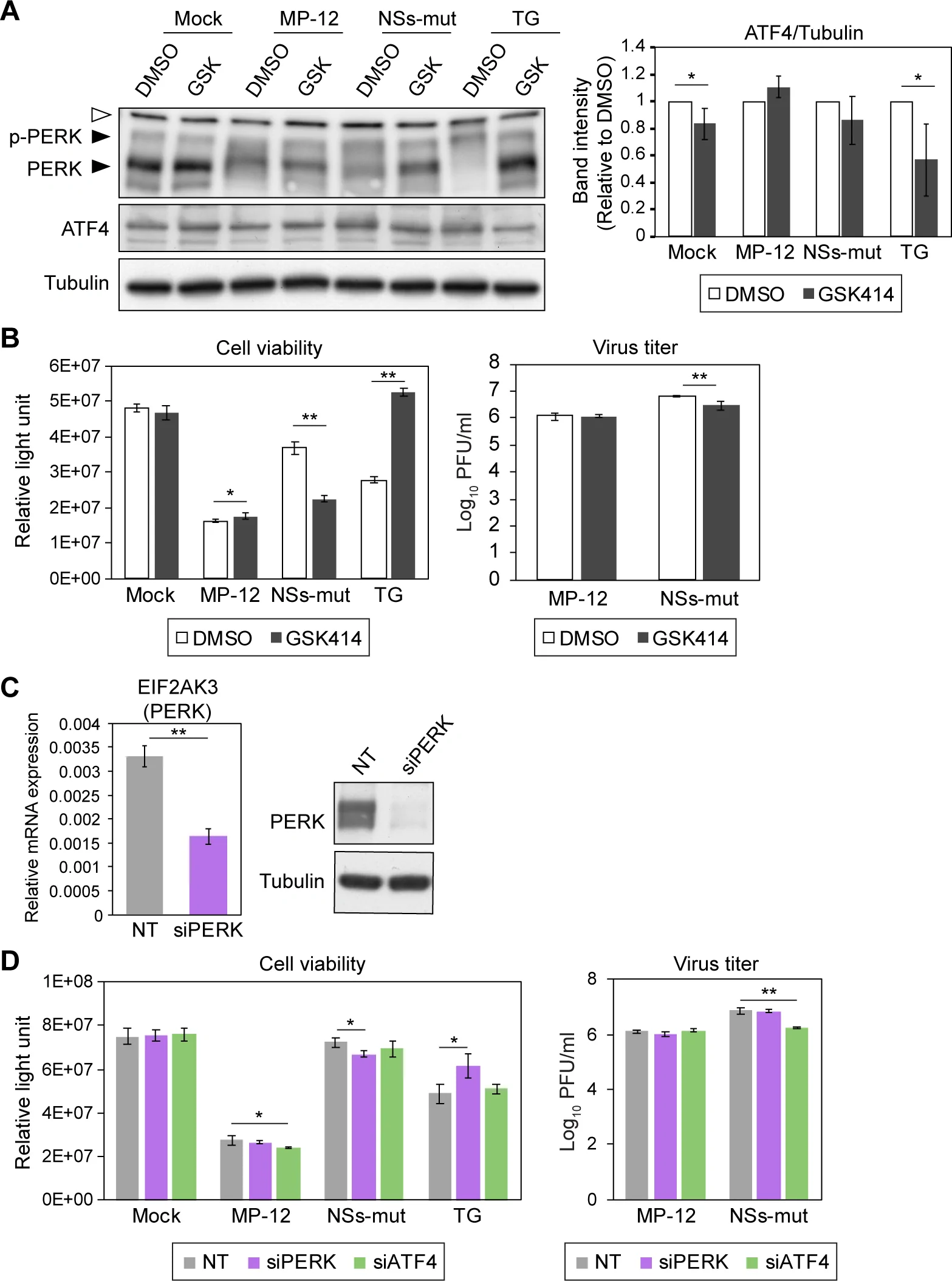
Inhibiting PERK reduces the viability of NSs-mut virus-infected cells. (**A**) Effect of PERK inhibitor on PERK phosphorylation. Vero E6 cells were infected with the indicated viruses at an MOI of 3 or left uninfected. TG-treated cells were used as a positive control. After virus inoculation, cells were treated with DMSO or GSK414 (0.3 μM). Cell lysates were harvested at 6 h p.i. and subjected to Western blot analysis. The open arrowhead represents non-specific signals. (**B**) Effect of PERK inhibitor on the viability of RVFV-infected cells. Virus infection and chemical treatment were performed as described in panel A. At 24 h p.i., DMSO, GSK414, and TG were added again without replacing the medium. At 48 h p.i., cells were subjected to an ATP assay. Culture supernatant was harvested at 48 h p.i. and subjected to a plaque assay to determine virus titer. (**C**) PERK depletion by siRNA transfection. Vero E6 cells were transfected with non-targeting (NT) siRNA or siPERK RNA. Cells were harvested at 48 h p.t. and analyzed by RT-qPCR and Western blot. (**D**) Effect of PERK depletion and ATF4 depletion on the viability of RVFV-infected cells. Vero E6 cells were transfected with the indicated siRNA 48 h prior to infection and infected with the indicated viruses or treated with TG. At 48 h p.i., cells were subjected to an ATP assay. Culture supernatant was collected at 48 h p.i. and subjected to a plaque assay. *P < 0.05, **P < 0.01.

GSK414 treatment reduced the amount of phosphorylated PERK and increased the amount of non-phosphorylated PERK in NSs-mut virus-infected cells at 6 h p.i. and in TG-treated cells (Fig. 6A), demonstrating GSK414-induced inhibition of PERK phosphorylation. Unexpectedly, we did not see clear evidence of GSK414-mediated inhibition of PERK phosphorylation in MP-12-infected cells. Nonetheless, we included MP-12-infected cells in subsequent analyses. GSK414 treatment also decreased the expression level of ATF4 in TG-treated cells (Fig. 6A), suggesting that the inhibition of PERK phosphorylation negated ATF4 expression. In contrast, no significant difference in ATF4 expression was observed between DMSO and GSK414 treatments in NSs-mut virus-infected cells.

We next analyzed the effect of GSK414 on the viability of infected cells and infectious virus production at 48 h p.i. We used TG-treated cells as a positive control. GSK414 treatment significantly decreased viability of NSs-mut virus-infected cells (Fig. 6B, left panel), suggesting that activation of PERK pathway has a pro-survival effect on NSs-mut virus-infected cells. In contrast, GSK414 treatment significantly increased the viability of TG-treated cells, suggesting a pro-death effect of the PERK pathway activation. GSK414 treatment also reduced the NSs-mut virus titers by approximately 60% (Fig. 6B, right panel). These data show that inhibiting the PERK pathway by GSK414 decreased cell viability and production of infectious viruses in NSs-mut virus-infected cells. Although GSK414 treatment slightly increased viability of MP-12-infected cells, its mechanism remains unknown since GSK414 did not inhibit PERK phosphorylation in MP-12-infected cells.

To further investigate the role of the PERK-ATF4 axis in the survival of RVFV-infected cells, we examined the effect of PERK or ATF4 depletion on the viability of infected cells. PERK depletion by siRNA transfection was confirmed by RT-qPCR and Western blot analysis (Fig. 6C). PERK depletion decreased the viability of NSs-mut virus-infected cells, while it increased the viability of TG-treated cells (Fig. 6D, left panel). These data suggest that PERK has a pro-survival effect in NSs-mut virus-infected cells and a pro-death effect in TG-treated cells. The data obtained from the PERK depletion study were consistent with those obtained from the GSK414 treatment study, yet the pro-survival effect of PERK depletion on NSs-mut virus infection was less pronounced than the effect mediated by the GSK414 treatment. The viability of TG-treated cells was unaffected by ATF4 depletion, suggesting that cell viability in TG-treated cells is not affected by the PERK-ATF4 axis and is controlled by other pathways downstream of PERK. ATF4 depletion also did not change the viability of NSs-mut virus-infected cells but decreased infectious virus production (Fig. 6D, right panel). Although ATF4 expression was already inhibited in MP-12-infected cells (Fig. 3), further ATF4 depletion decreased the viability of MP-12-infected cells.

## DISCUSSION

We investigated the impact of NSs-mediated transcription suppression on host gene expression by comparing the transcriptomes of Vero E6 cells infected with MP-12 to those infected with NSs-mut virus. MP-12 NSs differs from the NSs of WT RVFV strain ZH501 by one amino acid (59), and MP-12 NSs retains the molecular functions of WT RVFV NSs (29). In a mouse model, MP-12 is primarily attenuated by mutations in the L and M segments (60), suggesting that MP-12 NSs and WT RVFV NSs play a similar role in viral pathogenesis. Therefore, MP-12 is a suitable model for studying the molecular function of RVFV NSs. NSs suppresses host transcription in RVFV-infected cells (24), whereas approximately 70% of genes that are differentially expressed in MP-12-infected cells are upregulated compared to uninfected cells (Fig. 1A). The data suggest that NSs downregulates a limited number of host genes at 10 h p.i. and that the expression of many host genes is affected by the complex interplay between host cells and the virus.

**Fig 7:**
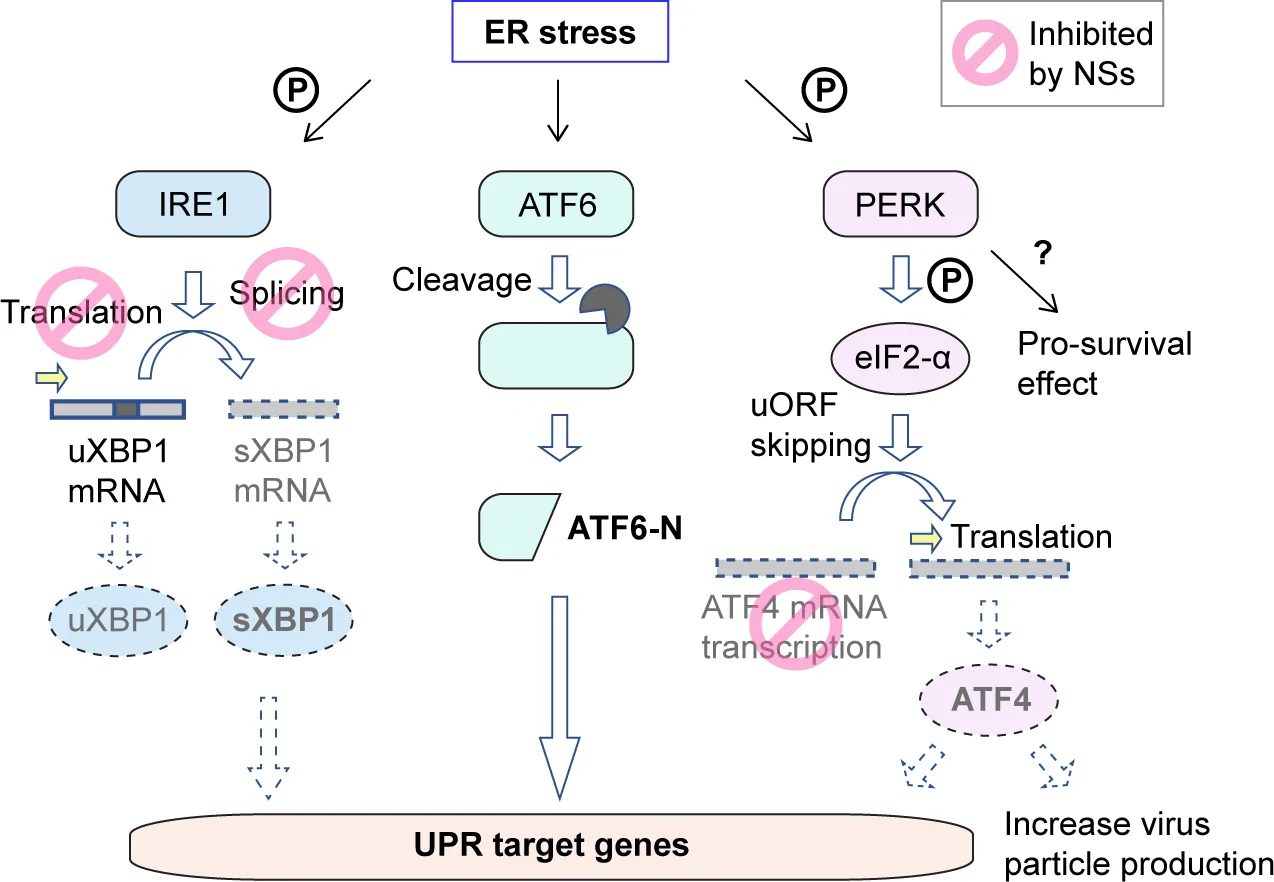
Graphical summary of NSs-mediated UPR inhibition. MP-12 infection activates all branches of UPR but inhibits the IRE1 and PERK branches via NSs-mediated suppression of XBP1 and ATF4.

Both MP-12 and NSs-mut virus activated all three ER stress sensors. We further showed that MP-12, but not NSs-mut virus, inhibited expression of XBP1 and ATF4, each of which acts downstream of the IRE1 pathway and PERK pathway, respectively. Based on the data obtained from the present study, Fig. 7 illustrates our model of NSs-induced UPR inhibition. Several viruses belonging to Bunyavirales induce ER stress and UPR (61–64). For example, severe fever with thrombocytopenia syndrome virus (SFTSV), which belongs to the same *Phlebovirus* genus as RVFV, induces ER stress through expression of Gn/Gc glycoproteins (63). RVFV Gn/Gc glycoproteins, which accumulate in the ER-Golgi compartment, may also serve as an inducer of the ER stress in infected cells. Unlike RVFV, SFTSV activates all three branches of the UPR, demonstrating that even closely related phleboviruses may regulate the UPR differently.

MP-12 suppressed expression of both uXBP1 and sXBP1 proteins (Fig. 2B), and the inhibition of sXBP1 expression was caused by the NSs-mediated suppression of XBP1 mRNA splicing (Fig. 2D). Others reported that RVFV infection alters splicing patterns of host mRNAs, including RIOK3 mRNA (65–67), although this alteration of splicing pattern is NSs independent (67). As XBP1 mRNA splicing occurs in the cytoplasm (45) and NSs suppresses mRNA export (44), we explored whether the NSs-mediated suppression of mRNA export is responsible for inhibition of XBP1 mRNA splicing. Our data suggest that the inhibition of XBP1 splicing induced by NSs is not due to NSs-mediated inhibition of mRNA export (Fig. S2). Inhibition of XBP1 mRNA splicing was also reported in SARS-CoV (68) and herpes simplex virus 1 (69). For the latter virus, viral protein UL41 suppresses IRE1 RNase activity, leading to inhibition of XBP1 mRNA splicing (69). It is possible that NSs suppresses the expression of host proteins required for XBP1 mRNA splicing. Further study is needed to clarify how NSs inhibits the splicing of XBP1 mRNA and the accumulation of the uXBP1 protein. Although the biological significance of the XBP1 inhibition in RVFV infection remains unknown, XBP1 regulates expression of inflammatory cytokines and ISGs (70, 71). This suggests that the NSs-mediated XBP1 inhibition may benefit RVFV by suppressing host antiviral responses.

While both MP-12 and NSs-mut virus induced phosphorylation of PERK and eIF2α, only MP-12 inhibited ATF4 expression by inhibiting ATF4 mRNA transcription (Fig. 3B). The results suggest that ATF4 is one of the targets of NSs-mediated host transcription suppression. In contrast, NSs-mut virus infection resulted in increased accumulation of both ATF4 mRNA and ATF4 protein (Fig. 3A and B). Since previous studies reported that ER stress increases ATF4 mRNA levels (72–74), it is likely that induction of ER stress in NSs-mut virus-infected cells upregulates ATF4 mRNA transcription. It has been reported that sXBP1 can increase ATF4 mRNA transcription by directly binding to the promoter region of the ATF4 gene (75). However, depletion of XBP1 did not influence ATF4 mRNA levels in our experiment (Fig. 4A).

We examined the roles of ATF4 and XBP1 in upregulation of several UPR genes in RVFV-infected cells. In NSs-mut infected cells, ATF4 contributed to upregulation of the HMOX1 gene, and XBP1 contributed to the upregulation of the SYVN1, HSPA5, and CRBPB genes (Fig. 5A). The PPP1R15A gene was also upregulated in NSs-mut virus-infected cells. However, neither ATF4 nor XBP1 affected the upregulation of this gene (see Fig. 5A), suggesting that other transcription factors related to ER stress induced PPP1R15A gene expression. Transcriptome analysis suggested that MP-12 inhibited expression of CEBPB mRNA and CEBPG mRNA, encoding transcription factors C/EBP-β and C/EBP-γ, respectively (Table 3). This downregulation of CEBPB mRNA was independent of ATF4 and XBP1 (Fig. 5A); hence, transcription of CEBPB mRNA could also be the direct target of NSs-mediated general transcription inhibition.

Our data suggest that ATF4 plays a key role in upregulating expression of the HMOX1 gene, which encodes the antioxidant HO-1 during NSs-mut virus infection (Fig. 5A). HO-1 is known to inhibit the replication of several viruses (76). Additionally, HO-1 stimulates expression of ISGs by interacting with IRF3 in influenza A virus-infected cells (77). The NSs-mut virus induced higher levels of many ISGs than MP-12 in Vero E6 cells (Table S2), likely through activation of non-canonical IFN-independent ISG induction pathways (78). It is possible that the higher HO-1 accumulation contributed to some of the ISG expression in NSs-mut virus-infected cells. If so, the NSs-mediated HO-1 suppression would inhibit ISG expression and would be beneficial for viral replication in animal hosts.

MP-12 is more cytotoxic than the NSs-mut virus in Vero E6 cells, despite both viruses replicating at similar levels (29). As MP-12 inhibited IRE1 and PERK UPR pathways, it is likely that NSs-mediated UPR inhibition prevents cells from recovering from ER stress, leading to the activation of a cell death pathway. Indeed, IRE1 phosphorylation levels remained high in MP-12-infected cells throughout the course of infection (Fig. 2A). While CHOP is a well-known apoptosis inducer under ER stress (58), the levels of DDIT3 mRNA, which encodes CHOP, were similar between MP-12-infected cells and NSs-mut virus-infected cells (Fig. 5A), suggesting that CHOP may not contribute to the higher cytotoxicity in MP-12-infected cells. Our study suggested that PERK phosphorylation contributes to cell survival in NSs-mut virus infection as GSK414 treatment decreased cell viability (Fig. 6B). Because PERK depletion had the same effect as GSK414 treatment (see Fig. 6D), it is less likely that the effect of GSK414 treatment on cell viability was an off-target effect, which was previously reported (79). The effect of PERK depletion on cell viability was smaller than GSK treatment in NSs-mut virus-infected cells (Fig. 6B and D). This potentially means that siRNA transfection process itself affected cell survival pathways.

PERK has several substrates, including eIF2α, NRF2, p38, and forkhead box O (80, 81). Because NSs-mut virus infection induced ATF4 expression, we examined the contribution of the PERK-eIF2α-ATF4 axis to cell survival in NSs-mut virus-infected cells. In NSs-mut virus-infected cells, ATF4 depletion did not affect cell viability (Fig. 6D), and GSK414 treatment significantly increased cytotoxicity without affecting ATF4 expression (Fig. 6A), suggesting that ATF4 was not responsible for the pro-survival effect of PERK. As ATF4 depletion reduced the replication of the NSs-mut virus (see Fig. 6D), ATF4 depletion-induced inhibition of virus replication may have affected cytotoxicity. We also investigated whether the other proteins, whose expression is increased by eIF2α phosphorylation, contribute to cell survival in NSs-mut virus-infected cells by using an inhibitor of the integrated stress response (ISRIB) to inhibit eIF2α phosphorylation. However, ISRIB did not inhibit eIF2α phosphorylation in either MP-12-infected cells or NSs-mut virus-infected cells (data not shown). Further studies are underway to understand how PERK activation promotes cell survival in RVFV-infected cells.

Our study suggested that ATF4 expression has a positive effect on virus particle production (Fig. 4C). The reduction in infectious virus production in ATF4-depleted cells was greater at 16 h p.i. than at 10 h p.i. (Fig. 4D and E). This is potentially due to the highest levels of infectious virus being present around 16 h p.i. under high MOI conditions (82). The positive impact of ATF4 expression on virus production raises the question of why NSs inhibits ATF4 expression. Interestingly, ATF4 depletion markedly decreased the viral particle production of both MP-12 and NSs-mut virus (Fig. 4C), while the production of infectious particles was only modestly affected (Fig. 4D). These results suggest that ATF4 depletion primarily reduces the production of non-plaque-forming virus particles. Because RVFV helical viral ribonucleoprotein complexes can trigger the induction of type I IFNs (83), infection of non-plaque-forming RVFV may trigger host antiviral responses, preventing efficient replication and spread of infectious viruses in mammalian hosts. Therefore, reducing the production of non-plaque-forming RVFV by inhibiting ATF4 expression may benefit the virus *in vivo*. ATF4 facilitates protein trafficking in the ER-Golgi compartment by upregulating genes related to this process (84). It is therefore conceivable that ATF4 depletion induces the retention of Gn/Gc in the ER and prevents their efficient translocation to the virus budding site in the Golgi apparatus, leading to a reduction in the formation of virus particles.

## MATERIALS AND METHODS

### Cells and virus

BSRT7/5 cells were maintained in Glasgow’s minimum essential medium (MEM) containing 10% fetal bovine serum (FBS), 10% tryptose phosphate broth, MEM amino acid solution, and 1 mg/mL of G418 (Gibco). Green monkey (*Chlorocebus sabaeus*)-derived Vero E6 cells were maintained in Dulbecco’s modified Eagle medium (Gibco) containing 5% FBS. All culture media were supplemented with 100 units/mL of penicillin and 100 µg/mL of streptomycin.

An attenuated RVFV strain, MP-12 (28), and NSs-mut virus, MP-12 carrying R16H and M250K mutations in NSs (29), were generated by a reverse genetics system (85) with modifications. Briefly, BSRT7/5 cells were transfected with T7-driven plasmids encoding viral proteins, L and N, and viral L, M, and S RNAs by using TransIT LT1 (Mirus bio). Culture supernatant was harvested on day 5 post-transfection and inoculated into Vero E6 cells for further amplification (P1 stock virus). Infectious doses of the P1 stock viruses were determined by standard plaque assay using Vero E6 cells.

### RNA-seq analysis

Vero E6 cells were infected with virus at an MOI of 3 and harvested in Trizol reagent at 10 h p.i. Two biological replicates were used for the subsequent analyses. Total RNA was extracted from the infected cells and subjected to poly-A mRNA selection using oligo-dT bound magnetic beads (NEB). After RNA fragmentation, cDNAs were synthesized using random primers. Libraries were synthesized by NEBNext Ultra II Directional RNA Library Prep Kit (NEB) for paired-end 75 bp on an Illumina NextSeq550 platform (Illumina). Base calling was performed using bcl2fastq (Illumina) and aligned to *Chlorocebus sabaeus* genome using STAR (86). Differential expression analysis between MP-12- and NSs-mut virus-infected samples was performed by the DESeq2 package, and pathway analysis was performed by QIAGEN Ingenuity Pathway Analysis (QIAGEN Inc., https://digitalinsights.qiagen.com/IPA) (87) with a threshold of *P* < 0.05 and log_2_ fold change of >1.5. The volcano plot was generated using the web app VolcaNoseR (88). For canonical pathway analysis, the *P* value cutoff was set below 0.05. The *z*-score cutoff for significantly activated or inhibited pathways was set above 2 or below −2, respectively.

### Purification of virus particles

The extracellular viruses released into the supernatant of infected cells were purified using sucrose gradient centrifugation, as previously described (89), with a modification. After removing cell debris by low-speed centrifugation, culture supernatant was layered on top of a step sucrose gradient consisting of 20%, 30%, 50%, and 60% (wt/vol) and centrifuged for 18 h at 26,000 rpm at 4°C using a Beckman SW28 rotor. Virus particles at the interface of 30% and 50% sucrose were collected and pelleted down through a 20% sucrose cushion at 38,000 rpm for 2 h at 4°C using a Beckman SW41 rotor.

### Western blot analysis

Western blot analysis was performed as previously described (90), except that ECL Plus Western Blotting Substrate (Thermo Fisher Scientific) and SuperSignal West Femto (Thermo Fisher Scientific) were used for detection. For detection of phosphorylated protein, a phosphatase inhibitor (Cell Signaling Technology) was added to the sample buffer. For primary antibodies against phosphorylated proteins, bovine serum albumin and Tris-buffered saline with 0.01% Tween 20 were used for blocking and washing, respectively. The following antibodies were used for Western blot analyses: anti-Gn (4D4 mouse monoclonal) (91), anti-GST-N (rabbit polyclonal) (92), anti-L (L434 rabbit polyclonal) (93), anti-NSs (rabbit polyclonal) (94), anti-IRE1alpha (Santa Cruz Biotechnology, sc-390960), antiphospho-IRE1α (Thermo Fisher Scientific, PA1-16927), anti-XBP1 (Abcam, ab220783), anti-PERK (Proteintech, 24390-1-AP), anti-eIF2α (Cell Signaling Technology, 9721), antiphospho-eIF2α (Cell Signaling Technology, 3597), anti-ATF4 (Santa Cruz Biotechnology, sc-390063), anti-ATF6 (Proteintech, 24169-1-AP), anti-GCN2 (Cell Signaling Technology, 3302), antiphospho-GCN2 (Proteintech, 82835-3-RR), anti-JNK, antiphospho-JNK (Proteintech, 60666-1-1g), anti-HO-1 (Cell Signaling Technology, 5853), anti-SYVN1 (Cell Signaling Technology, 14773), anti-C/EBP-β (Cell Signaling Technology, 43095), anti-CHOP (Cell Signaling Technology, 2895), anti-BiP (Cell Signaling Technology, 3177), anti-GADD (Proteintech, 3410449-1-AP), anti-α-tubulin (Cell Signaling Technology, 3873), anti-PABP (Abcam, ab21060), goat antirabbit IgG (Cell Signaling Technology, 7074), and goat antimouse IgG (Cell Signaling Technology, 7076). The data represent one of three biological replicates. Band intensities of three biological replicates were analyzed using ImageJ and were used for statistical analysis. An unpaired Student’s *t*-test was used to analyze the statistical difference between the samples.

### RT-PCR for detection of spliced and unspliced XBP1 mRNA

Total RNA was extracted from cells using Trizol reagent (Thermo Fisher Scientific) and subjected to cDNA synthesis using the SuperScript III First-Strand Synthesis System (Thermo Fisher Scientific) with random primers. Primers for amplification of both spliced and unspliced XBP1 were designed based on a sequence of *Chlorocebus sabaeus* XBP1 gene (XM_007975273). PCR was performed using KOD Xtreme Hot Start DNA Polymerase (Sigma-Aldrich) with forward primer (5′ cagcgcctggggatggatgc 3′) and reverse primer (5′ aagggaggctggtaaggaac 3′). Primers were designed based on a previous publication (95) with modifications.

### RT-qPCR

RNA extraction and cDNA synthesis were performed as described above in RT-PCR. Primer sequences used for the qPCR are shown in Table S3. Duplicate qPCR was performed using SsoAdvanced Universal SYBR Green Supermix (Bio-Rad) for each sample. The average of the two wells was used for analysis. The relative standard curve method was used for relative quantification of RNA. The relative amount of target RNA was normalized by the amount of 18S ribosomal RNA. Data represent the average of three biological replicates. The statistical difference was analyzed using an unpaired Student’s *t*-test.

### siRNA treatment

For depletion of ATF4, XBP1, and PERK (EIF2AK3) expression, siRNA targeting human ATF4, XBP1, and EIF2AK3 genes (On-target Plus SmartPool, Horizon Discovery), each containing four siRNAs, was used. Since we used siRNAs designed for human genes, the sequence of XBP1 and ATF4 siRNAs had a total of one nucleotide and five nucleotide mismatches, respectively, when aligned to the *Chlorocebus sabaeus* XBP1 and ATF4 sequences. Cells were transfected with 37.5 nM of siRNA using TransIT-siQuest (Mirus Bio) 2 days prior to virus infection.

### Hydrogen peroxide measurement

Hydrogen peroxide levels were measured by the ROS-Glo H_2_O_2_ Assay Kit (Promega). Cells were infected with the indicated viruses at an MOI of 3 or left uninfected and incubated with H_2_O_2_ substrate, which reacts with H_2_O_2_ and generates luciferin precursor, 4 h prior to detection. A detection solution was added to the cells at the indicated time points, and luminescent signals were measured by a luminometer. Data represent the average of three biological replicates. The statistical difference was analyzed using an unpaired Student’s *t*-test.

### Cell viability assay

Cellular ATP was measured using Viral ToxGlo (Promega). For GSK414 treatment, Vero E6 cells were grown in 96-well plates and inoculated with the indicated viruses or left uninfected. After incubation with the virus for 1 h, culture medium containing DMSO or 0.3 μM of GSK2606414 (MilliporeSigma) was added to the wells. Control wells were also treated with 400 nM of TG. At 24 h p.i., DMSO or 0.3 μM of GSK2606414 was further added to the wells without replacing the medium. ATP detection reagent was added to the wells at 48 h p.i. After incubation for 15 min at room temperature, luminescence was measured. For siRNA transfection, Vero E6 cells were grown in 96-well plates and transfected with non-targeting siRNA or siRNA targeting PERK and ATF4. At 48 h post-transfection, cells were inoculated with the indicated viruses and left uninfected or treated with TG. ATP was measured at 48 h p.i. as described above.

### Northern blot

VeroE6 cells were infected with the indicated viruses at an MOI of 3. Total RNA was extracted at 6 and 10 h post-infection using TRIzol reagent (Invitrogen). Northern blot was performed as described previously (96) using digoxigenin-labeled RNA probes that bind to viral genomic sense L, M, and S RNAs.

### Indirect immunofluorescence assay

Cells were fixed by 4% paraformaldehyde and permeabilized by 0.1% Triton X-100. After blocking with 1% BSA, cells were incubated with primary antibodies followed by fluorescent-conjugated secondary antibodies. Images were captured by the ImageXpress confocal microscope (Molecular Devices) and analyzed by ImageJ software.

## Data Availability

RNA-seq data are deposited in NCBI Gene Expression Omnibus under accession number GSE344700.

## SUPPLEMENTAL MATERIAL

Supplemental material jvi.01233-26-s0001.pdf Fig. S1 and S2; Tables S1 to S3.

ASM does not own the copyrights to Supplemental Material that may be linked to, or accessed through, an article. The authors have granted ASM a non-exclusive, world-wide license to publish the Supplemental Material files. Please contact the corresponding author directly for reuse.
