# Supplementary material for: Rift Valley fever virus virulence factor NSs targets ATF4- and XBP1-driven unfolded protein response
Source: J Virol. 2026 Sep 24;100(10):e01233-26. doi: 10.1128/jvi.01233-26 (PMC13712871; doi:10.1128/jvi.01233-26)
Supplement: Supplementary file 1 [file jvi.01233-26-s0001.pdf]

Fig. S1

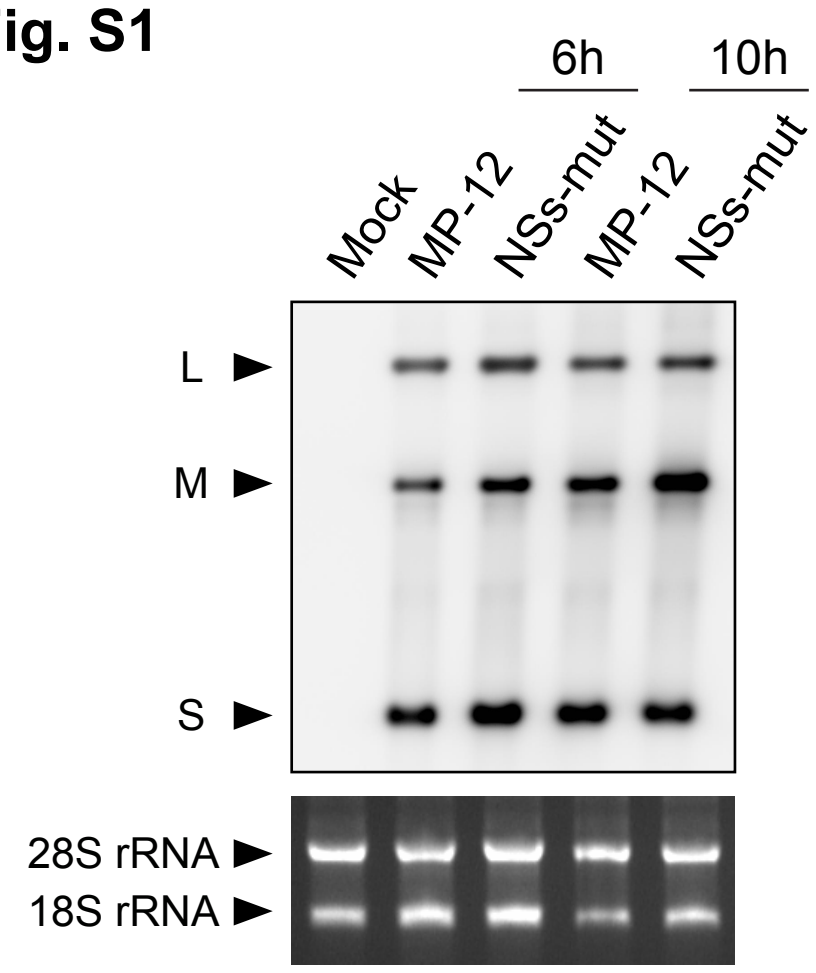

Supplemental Fig. 1. Viral RNA levels at 6 and 10 h p.i. Vero E6 cells were infected with the indicated virus at an MOI of 3. Total RNA was extracted at 10 h p.i. and subjected to Northern blot.

Fig. S2

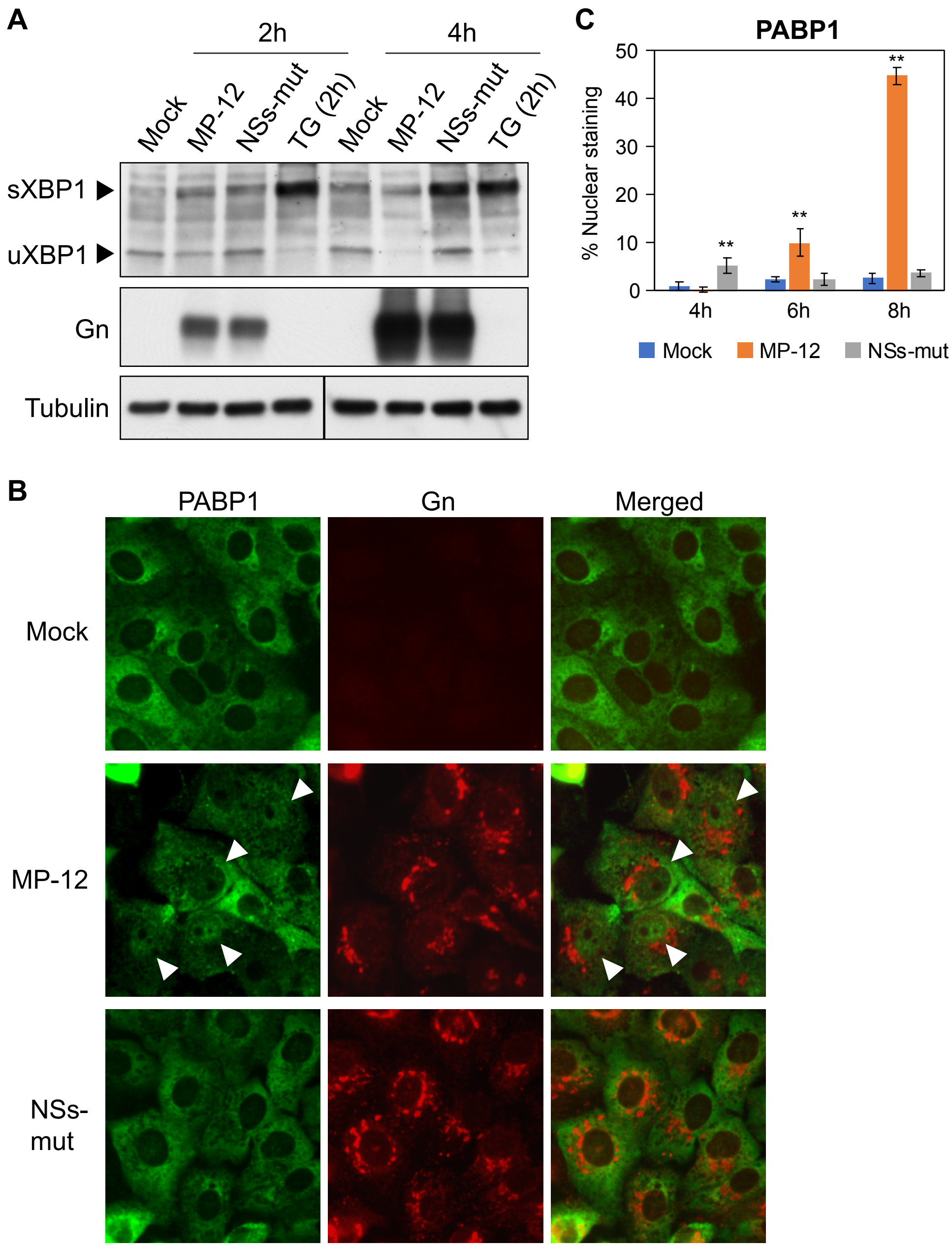

Supplemental Fig. 2. Correlation of NSs-mediated XBP1 inhibition and inhibition of nuclear transport. (A) Expression of sXBP1 and uXBP1 early in infection. Vero E6 cells were infected with the indicated virus at an MOI of 3. Uninfected cells and cells treated with TG for 2 h were used as controls. Cell lysates were harvested at 2 and 4 h p.i., and analyzed by Western blot using anti-XBP1 antibody, which detects both sXBP1 and uXBP1, anti-Gn antibody and anti-Tubulin antibody. (B) Subcellular localization of PABP1 in infected cells. Vero E6 cells were mock-infected or infected with the indicated virus at an MOI of 3 and subjected to immunofluorescence assay by using antibodies against RVFV Gn and PABP1 at 8 h p.i.. (C) Percentage of cells showing nuclear PABP1 staining among Gn-positive cells. **P<0.01.

Table S1. Top 10 upregulated genes in RVFV-infected cells compared with uninfected cells.

	Symbol	Log_2 Fold Change	padj	Gene name
MP-12 vs Mock	TNFAIP3	6.7	0	TNF alpha induced protein 3
	NFKBIA	5.6	0	NFKB inhibitor alpha
	IFIT3	5.2	0	Interferon induced protein with tetratricopeptide repeats 3
	NFKBIZ	5	0	NFKB inhibitor zeta
	IFIH1	4.4	0	Interferon induced with helicase C domain 1
	ATF3	4.4	0	Activating transcription factor 3
	IRF1	4.3	0	Interferon regulatory factor 1
	DHX58	4.2	0	DExH-box helicase 58
	FRMD6	3.7	0	FERM domain containing 6
	HIVEP2	3.4	0	HIVEP zinc finger 2
NSs-mut virus vs Mock	IFIT3	5.6	0	Interferon induced protein with tetratricopeptide repeats 3
	IFIH1	4	0	Interferon induced with helicase C domain 1
	NFKBIA	3.8	0	NFKB inhibitor alpha
	TNFAIP3	3.4	0	TNF alpha induced protein 3
	HERPUD1	3.2	0	Homocysteine inducible ER protein with ubiquitin like domain 1
	VCAM1	3	0	Vascular cell adhesion molecule 1
	ZC3HAV1	2.7	0	Zinc finger CCCH-type containing, antiviral 1
	HSPA5	2.5	0	Heat shock protein family A (Hsp70) member 5
	PPP1R15A	2.3	0	Protein phosphatase 1 regulatory subunit 15A
	TNIP1	2.2	0	TNFAIP3 interacting protein 1

Table S2. Upregulated ISGs in RVFV-infected cells.

Symbol*	Log_2 Fold Change MP-12 vs Mock	Log_2 Fold Change NSs-mut vs Mock
IFIT3	5.184	5.649
IFIH1	4.423	4.014
ZC3HAV1	3.202	2.747
IFIT2	6.152	6.483
IFIT1	4.617	6.684
ISG15	3.876	5.381
IRF1	4.295	2.655
DDX60	6.503	6.982
RIGI	1.17	2.822
ISG20	3.052	3.716
MX1	3.274	5.496
DDIT4	2.959	2.423
IFIT5	2.511	2.682
IFI44	7.106	8.152
OAS1	0.837	3.866
OAS2	1.125	2.695
MX2	4.977	6.264
RSAD2	1.381	2.27
GBP1	4.592	4.159
IFIT1B	4.372	4.881

*The order of the genes begins with the gene of highest significance (-p-value) in NSs-mut virus-infected cells. Gray boxes indicate the numbers that were not significant vs mock.

Table S3. Sequence of qPCR primers.

	Sequence 5' to 3'
ATF4-F	TTCTCCAGCGACAAGGCTAAGG
ATF4-R	CTCCAACATCCAATCTGTCCCG
XBP1-total-F	TGAAAAACAGAGTAGCAGCTCAGA
XBP1-total-R	CCCAGGCGCTGTCTTAACTC
XBP1-unspliced-F	CAGACTACGTGCACCTCTGCAG
XBP1-unspliced-R	CTGGGTCCAAGTTGTCCAGAATG
18S rRNA-F	CCGGTACAGTGAAACTGCGAATG
18S rRNA-R	GTTATCCAAGTAGGAGAGGAGCGAG
HMOX1-F	CCAGGCAGAGAATGCGGAGTTC
HMOX1-R	AAGACTGGGCTCTCCTTGTTGC
HSPA5-F	CTGTCCAGGCTGGTGTGCTCT
HSPA5-R	CTTGGTAGGCACCACTGTGTTC
PPP1R15A-F	TCCGACTGCAAAGGCGGCTCA
PPP1R15A-R	CAGCCAGAAAATGGACAGTGAC
DDIT3-F	GCTGGAAAGCAGCGCATGAA
DDIT3-R	ACGAGTCGCCTCTACTTCCC
SYVN1-F	CCAACATCTCCTGGCTCTTTCAC
SYVN1-R	GTCAGGATGCTGTGATAGGCAT
CEBPB-F	CAAGAAGACAGTGGACAAGC
CEBPB-R	AGCTGCTCCACCTTCTTCTG
